# Inhibitory Effects on Clinical Isolated Bacteria and Simultaneous HPLC Quantitative Analysis of Flavone Contents in Extracts from *Oroxylum indicum*

**DOI:** 10.3390/molecules24101937

**Published:** 2019-05-20

**Authors:** Patchima Sithisarn, Piyanuch Rojsanga, Pongtip Sithisarn

**Affiliations:** 1 Department of Veterinary Public Health, Faculty of Veterinary Medicine, Kasetsart University, Bangkok 10900, Thailand; fvetphs@ku.ac.th; 2 Department of Pharmaceutical Chemistry, Faculty of Pharmacy, Mahidol University, Bangkok 10400, Thailand; piyanuch.roj@mahidol.ac.th; 3 Department of Pharmacognosy, Faculty of Pharmacy, Mahidol University, Bangkok 10400, Thailand

**Keywords:** *Oroxylum indicum*, baicalin, baicalein, chrysin, *Staphylococcus intermedius*, *Streptococcus suis*, broth micro-dilution assay, HPLC

## Abstract

*Oroxylum indicum* is a medicinal plant in Thailand, which has been used as a tonic and for the treatment of various diseases. Extracts from various parts of *O. indicum* were reported as promoting in vitro antioxidant and antibacterial effects. Phytochemical analysis suggested that this plant contained some flavones. *O*. *indicum* fruit and seed water and ethanol extracts and their major flavonoids including baicalein, baicalin, and chrysin were tested for in vitro antibacterial activities on four clinical isolated bacteria, namely, *Staphylococcus intermedius*, *Streptococcus suis*, *Pseudomonas aeruginosa*, and *β-Escherichia coli*, using a broth micro-dilution assay. The amounts of these three major flavonoids were also quantitatively analyzed using the high-performance liquid chromatographic (HPLC) method. *O. indicum* fruit ethanol extract from Nakhon Pathom province (OFNE) promoted the strongest antimicrobial activity against four clinical pathogenic bacteria, including *S. intermedius* (IC_50_ = 1.30 mg/mL), *S. suis* (13.59% inhibition at 7.81 mg/mL), *P. aeruginosa* (IC_50_ = 39.20 mg/mL), and *β-E. coli* (IC_50_ = 66.85 mg/mL). Baicalin showed high in vitro antibacterial effect to all tested bacteria. From the optimized and validated HPLC method, baicalin, baicalein, and chrysin contents in *O. indicum* extracts were 0.19 ± 0.00 − 9.45 ± 0.13, 0.14 ± 0.00 − 1.27 ± 0.02, and 0.02 ± 0.00 − 0.96 ± 0.02 g/100 g extract, respectively. Baicalin was found to be the major compound in *O. indicum* seed extract followed by baicalein, whereas chrysin was found in lower amounts than the amounts of the other two flavonoids in all *O. indicum* extracts.

## 1. Introduction

Ethnoveterinary medicine (EVM) is the traditional practice of using natural products, mainly plant extracts, to protect, treat, or support animal health [1]. The close association between humans and their pets and the high growth rate of productive animal industries has led to a high risk of zoonoses. These diseases can cause severe pathologies including septicemia and meningitis, which lead to the increase of antibiotic usage. However, with the realization of the antibiotic resistance crisis in humans and animals, as well as concerns about food safety and the quality of import–export food animal products, a limitation on antibiotic use is now being enforced. The uses for natural products and their active compounds are now of interest. Various secondary metabolites from plants such as volatile oils, alkaloids, phenolics, flavonoids, and anthraquiones were previously reported to promote antibacterial effects. *Oroxylum indicum* (L.) Vent. is a deciduous tree in the Bignoniaceae family. The mature fruit is both acrid and sweet, which promotes anti-helminthic and stomachic effects [2]. The seeds have been used as a purgative, while the seed paste is applied to the throat for the quick relief of tonsil pain [3]. Many flavonoids such as baicalein, biochanin A, oroxylin A, chrysin, apigenin, and their glycosides such as baicalein-7-*O*-diglucoside (oroxylin B), baicalein-7-*O*-glucoside, baicalein-6-*O*-glucoside (tetuin), and scutellarin-7-*O*-rutinoside were previously reported in the pods, seeds, and root bark of this plant [4,5,6,7,8,9,10]. The seeds and pods also contain alkaloids and triterpenes such as ursolic acid [6,11]. The seed oil is composed of caprylic, lauric, myristic, palmitic, palmitoleic, stearic, oleic, and linoleic acids [2]. The leaves and stem barks also contain some flavones and flavone glycosides as well as other phenolics such as anthraquinone, tannic acid and ellagic acid, alkaloids, and phytosterols [4,5,12,13,14].

Our previous reports revealed that ethanol extracts from *O. indicum* fruits exhibited intermediate and moderate in vitro antibacterial activities against *Staphylococcus intermedius* and *Streptococcus suis* determined by the disc diffusion method, whereas the seed ethanol extract showed strong in vitro antioxidant activity tested by the DPPH scavenging method. Phytochemical analysis from that study also suggested the presence of baicalein [15]. Another report from our previous study mentioned that the extracts from various plant parts of *O. indicum* such as the leaves, flowers, seeds, stalks, and extracts from tissue-cultured plants and callus exhibited high in vitro antioxidant activities determined by a DPPH scavenging assay with high total phenolic and total flavonoid contents. The phytochemical profiles analyzed by TLC and LC-MS showed the presence of some flavones including baicalin, baicalein, and chrysin [16]. As is known today, flavones, the major and unique compounds in *O. indicum*, harbor many significant medicinal activities for modern medicinal usages, as our previous report correspondingly suggested with respect to in vitro antibacterial activities of the extracts from the fruits and seeds. However, there is still no report concerning the active compounds and the efficacy of use for this plant against the pathogenic bacteria that can cause lethal meningococcal meningitis and soft tissue bacterial meningitis. The clinical bacterial isolates were selected in order to display the clinical implications which will be very useful for both veterinary medicine and human medicine. In addition, there is no analytical method to simultaneously and quantitatively analyze these flavones. Therefore, in order to investigate the effectiveness of the extracts and clarify the active constituents’ ability to inhibit clinical isolated bacteria, six extracts and three major flavones from *O. indicum*, namely, baicalin, baicalein, and chrysin, were determined for the half maximal inhibitory activity (IC_50_) and the ninety-seven percent inhibitory concentrations (IC_97_) on four clinical isolated bacteria using a broth micro-dilution assay—*Staphylococcus intermedius*, *Streptococcus suis*, *Pseudomonas aeruginosa*, and *β-Escherichia coli*. The high-performance liquid chromatographic (HPLC) method was optimized and validated for simultaneous quantitative analysis of three major flavone contents in *O. indicum* extracts.

## 2. Results

As shown in Table 1, ethanol extract from the *O. indicum* fruits collected from Nakhon Pathom province (OFNE) exhibited the strongest inhibitory effects on the selected clinical isolated bacteria. The half maximal inhibitory concentrations (IC_50_) of this extract to inhibit *S. intermedius*, *S. suis*, *P. aeruginosa*, and *β-E. coli* were 1.30, % inhibition = 13.59 at the concentration of 7.81 mg/mL, 39.20, and 66.85 mg/mL, respectively. Ethanol extract from *O. indicum* fruits collected from Chiang Rai province (OFCE) also exhibited an inhibitory effect on *S. intermedius* with IC_50_ of 1.96 mg/mL. *O. indicum* fruit water extracts and seed ethanol and water extracts promoted lower inhibitory effects on the tested bacteria.

**Figure 1:**
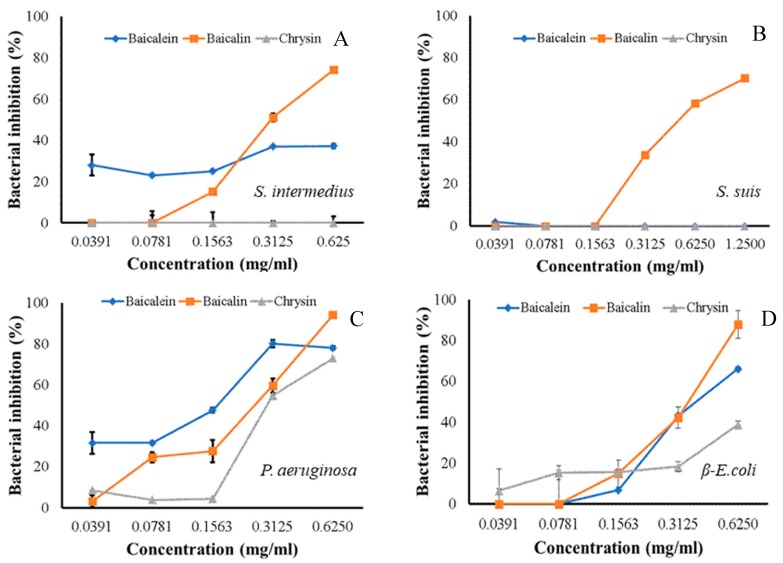
Dose-dependent manners of baicalein, baicalin, and chrysin inhibitory effects against four clinical isolated bacteria: *Staphylococcus intermedius* (**A**), *Streptococcus suis* (**B**), *Pseudomonas aeruginosa* (**C**), and *β-Escherichia coli* (**D**).

The ninety-seven percent inhibitory concentrations (IC_97_) of baicalin and baicalein to *S. intermedius* were 2.48 and 20,413 mg/mL, respectively, whereas chrysin did not exhibit any effect. The IC_97_ value of the baicalin to inhibit *S. suis* was 12.43 mg/mL, whereas baicalein and chrysin showed no effect. *P. aeruginosa* showed susceptibility to all three flavones, namely, baicalein, baicalin, and chrysin with IC_97_ values of 5.99, 1.83, and 1.81, mg/mL, respectively. *β-E. coli* displayed susceptibility with IC_97_ values of 1.86, 2.31, and 169.86 mg/mL for baicalein, baicalin, and chrysin, correspondingly (Table 2). Almost all substances with activities showed dose-dependent manners, aside from baicalein against *S. intermedius* that had a gradual increase in inhibition (Figure 1). From the results, baicalein, baicalin, and chrysin could be suggested as an active compound for antibacterial activities in *O. indicum* in a dose-dependent manner.

Linear correlations from 1.04–104, 1.08–108, and 1.12–112 μg/mL were obtained from baicalin, baicalein, and chrysin, respectively (Table 3). The *r*^2^ values for all three compounds were ≥0.9990, confirming the linearity of the method. From the investigation of peak purity, there was no indication of co-elution or impurities from all peaks of interest. The recoveries of all three concentrations of all compounds were close to theoretical amounts (% recoveries were in the range from 90 to 101%, %RSD < 2). Percentages of relative standard deviation (%RSD) of six standard baicalin, baicalein, and chrysin solutions analyzed in three different days (intermediate precision) were less than 5%, while the %RSD values for repeatability were not more than 2%. Therefore, the method can be regarded as precise. The validation parameters obtained from the optimized HPLC methods were acceptable [17]; therefore, this analytical HPLC method could be used for quantitative analysis of the three flavonoids in the *O. indicum* extracts. For system suitability parameters, tailing factors of all three standard compounds were less than 2, theoretical plate numbers were in the range of 10,000 to 90,000, %RSD values of the peak area were less than 2 and the standard deviation (SD) values of the retention times of each peak were less than 1, suggesting the suitability of the analytical method.

As shown in Table 4, the contents of baicalin, baicalein, and chrysin in all *O. indicum* extracts were determined using the validated HPLC method. All *O. indicum* extracts showed similar HPLC chromatograms with the peaks corresponding to baicalin, baicalein, and chrysin. From HPLC analysis, baicalin was found to be the major compound in all *O. indicum* extracts, followed by baicalein, whereas chrysin was found in the lowest amount among these flavonoids. The mature seed extracts (OSBD and OSBE) contained the highest amounts of baicalin at 9.03 ± 0.08 and 9.45 ± 0.13% *w*/*w* g extract, respectively, and high amounts of baicalein at 1.27 ± 0.02 and 0.91 ± 0.02% *w*/*w* g extract, respectively. The whole fruit extracts from Nakhon Pathom and Chiang Rai provinces prepared by maceration (OFNE and OFCE, respectively) also contained high amounts of baicalein and moderate amounts of baicalin, whereas OFND and OFCD that are fruit extracts from Nakhon Pathom and Chiang Rai provinces prepared using the decoction method contained low amounts of both baicalin and baicalein contents.

## 3. Discussion

From the results, extracts from the young whole fresh fruits of *O. indicum* exhibited a higher inhibitory effect on selected clinical isolated bacteria than the seed extracts, suggesting a higher accumulation of the active compounds in the fruits than the seeds. Ethanol extracts from *O. indicum* fruits also exhibited stronger inhibitory effects than the water extracts. These results correspond to our previous screening report, which suggested that ethanol extracts from the fruits of *O. indicum* exhibited a higher inhibitory effect on *S. intermedius* and *S. suis* than the water extract, whereas both seed ethanol and water extracts exhibited low inhibitory effects [10]. Three major flavones from *O. indicum* exhibited antibacterial effects on the tested bacteria. However, baicalin promoted the highest inhibitory effects, suggesting it is the main active compound in the extract. Our previous phytochemical analysis by LC-MS of extracts from various parts of *O. indicum* such as whole fruits, seeds, leaves, flowers, stalks, and pedicels suggested that the amounts of baicalin, baicalein, and chrysin in the whole fruit extract are high, especially the amount of baicalin, which is higher than in the other extracts from different plant parts [16]. Baicalin and baicalein also exhibited strong free radical scavenging effects when tested by a DPPH scavenging assay, whereas chrysin exhibited a lower inhibitory effect [16].

The extract from the fruits of *O. indicum* collected from Nakhon Pathom province (OFNE) exhibited the strongest antibacterial effects against all four tested pathogenic bacteria, namely, *Staphylococcus intermedius*, *Streptococcus suis*, *Pseudomonas aeruginosa*, and *β-Escherichia coli*, whereas the other five extracts promoted low inhibitory effects. Baicalin was found to be the active constituent for antibacterial activity, whereas baicalein and chrysin promoted a lower, inhibitory effect, except for *P. aeruginosa* for which chrysin and baicalein showed IC_97_ values of 1.81 and 5.99 mg/mL, respectively. However, the extracts which contained the highest baicalin contents were OSBD and OSBE (9.03 ± 0.08 and 9.45 ± 0.13% *w*/*w* of the extract, respectively), showing lower antibacterial effects to the selected pathogenic bacteria, except for OFCE which contained baicalin at 1.46% *w*/*w* of the extract and showed low IC_50_ value against *S. intermedius* of 1.96 mg/mL. From the results, high baicalin contents in OSBE and OSBD seemed to show some correlations to the in vitro antioxidant effects in the results of our previous study [10], but did not show good correlation to the in vitro antibacterial effects. This could be because there is a specific ratio or range of the active flavonoids in *O. indicum* extracts necessary to promote strong antibacterial effect or there could be other active constituents that promote antibacterial activity in *O. indicum* extracts. Moreover, a previous study reported that there is a fixed oil in the seeds of *O. indicum* [18]. This seed fixed oil could interfere with the antibacterial effects of flavonoids in the seed extracts.

With respect to the mechanism of action, it has been reported that there are two possible mechanisms related to the inhibitory effect on *Staphylococcus aureus* of the flavonoid-rich traditional Japanese medicine KRT, namely Keigairengyoto, a pharmaceutical-grade traditional Japanese (kampo) medicine comprised of seventeen crude drugs, direct bactericide and immune modulation [19], while another report has suggested that the mechanisms of the antibacterial action are forming a complex with proteins through non-specific forces such as hydrogen bonding and hydrophobic effects, as well as by covalent bond formation, which is related to their ability to inactivate microbial adhesins, enzymes, and cell envelope transport proteins [20]. Baicalein, which is the main compound in KRT, was also reported to have bactericidal effects on *S. aureus* [21]. The study also suggested that flavonoids circulate as inactive glucuronides and are converted to active aglycones in local inflammatory lesions, possibly by β-glucuronidase expressing macrophages, in which baicalin, a baicalein-7-*O*-glucuronide, is one of the key flavonoids mediating the antimicrobial effects [19]. However, there is a study indicating that flavones and isoflavones promoted lower diffusion abilities through the culture medium compared with flavanones and isoflavanones [22]. Normally, conjugation is a common detoxification reaction leading to the increased solubility of compounds, which is important for excretion [23]. Baicalin, a flavone glucuronide, has higher water solubility than its aglycone, baicalein, which could support its ability to be an antibacterial agent. Moreover, the extracts and major flavonoids exhibited stronger inhibitory effects on *S. intermedius* and *S. suis* as opposed to *P. aeruginosa* and *β-E. coli*. The results from this study also support the previous data that reported that apigenin-7-*O*-glucoside, which is a flavone glycoside, had evident antibacterial activity, specifically against Gram-positive bacteria [24]. Distinct from Gram-positive bacteria *Staphylococcus* and *Streptococcus* spp., recent studies have shown that Gram-negative bacteria were inhibited by baicalin through the inhibition of important virulence molecules and bacterial quorum sensing pathways. *P. aeruginosa* was reported to be inhibited by baicalin through controlling quorum sensing (QS)-controlled virulence factor and biofilm formation [25]. Additionally, baicalin was reported as interfering with lethal *Escherichia coli* (STEC) O157:H7 infections by inducing a Shiga-like toxin 2 (Stx2) to form inactive oligomers [26]. The differences in the mechanisms of flavonoids in inhibiting bacteria may expose distinctive patterns of inhibition and differences in dose-dependent manners.

From our study, OFNE could be a good source for antibacterial activity against four selected pathogenic bacteria, and all three flavonoids, namely, baicalin, baicalein and chrysin, could be used to control the quality of the extracts. The seeds of *O. indicum* are good sources to obtain high amounts of active flavonoids; extraction by maceration with 95% ethanol promoted extracts with higher amounts of these flavonoids than decoction. *O. indicum* whole fruits from Nakhon Pathom and Chiang Rai provinces contained flavonoid contents in the near ranges. Therefore, *O. indicum* fruit ethanol extracts could be further developed for pharmaceutical or medicinal purposes for their antibacterial effects.

## 4. Materials and Methods

### 4.1. Chemicals

Deionized water was obtained by using a water purification system from Thermo Scientific Co. (Waltham, MA, USA). Standard baicalin and chrysin, at a pharmaceutical grade, were purchased from TRC (North York, ON, Canada), and baicalein, at an analytical reference grade, was purchased from Sigma-Aldrich (St. Louis, MO, USA). DMSO was purchased from Sigma-Aldrich (St. Louis, MO, USA). Mueller-Hinton broth powder was purchased from Oxoid (Basingstoke, UK). Acetonitrile was purchased from Sigma-Aldrich (St. Louis, MO, USA), whereas phosphoric acid was purchased from Merck (Darmstadt, Germany).

### 4.2. Plant Materials

The fruits from *Oroxylum indicum* were separately collected from Chiang Rai and Nakhon Pathom provinces, whereas the mature seeds were purchased from a traditional herbal shop in Bangkok in 2015. Plant samples were cleaned and dried in a hot air oven (Memmert, Schwabach, Germany) at 60 °C, then ground using an electric mill (Ika-Werke, Staufen, Germany) (20 mesh sieve).

### 4.3. Bacteria and Reagents

Clinical isolates of *Staphylococcus intermedius*, *Streptococcus suis*, *Pseudomonas aeruginosa*, and *β-Escherichia coli* were obtained from the Microbiological Laboratory, Veterinary Diagnostic Center, Faculty of Veterinary Medicine, Kasetsart University, Nakhon Pathom, Thailand. The bacteria strains were isolated and characterized using differential bacterial culture and biochemical assays for clinical samples according to the standard method of Baron et al. [27]. *S. Suis*, *P. aeruginosa*, and *β-E. coli* were maintained in Microbank Cryovials and kept at −80 °C. *Staphylococcus intermedius* was maintained in skimmed milk at −40 °C until use. Blood agar was obtained from the Bacteriology Unit. Antibiotics amoxicillin, doxycycline, and gentamicin were purchased from Sigma-Aldrich (St. Louis, MO, USA).

### 4.4. Plant Extract Preparations

Each sample of *O. indicum* fruit and seed powder was separately extracted by maceration using 95% ethanol and decoction with distilled water (plant:solvent ratio 1:20 *w*/*v*). For decoction, each powdered plant sample was separately boiled with distilled water at 100 °C for 2 h and filtered, whereas for maceration, each powdered plant sample was separately macerated with 95% ethanol using an electric flask shaker (Wisd Laboratory Instruments, Wertheim, Germany) for 6 h. After the extract was kept for 12 h, the extraction solution was filtered. Each extraction process was repeated three times. The extraction solutions were then combined, filtered, and evaporated using a water bath to yield the dried extracts.

### 4.5. Bacteria Culture

Prior to sensitivity testing, each bacterial strain was maintained on a blood agar plate and incubated for 18–24 h at 37 °C.

### 4.6. Flavonoid Preparation

Flavonoids were dissolved in DMSO to reach the concentrations ranging between 0.0391 and 5 mg/mL and stored at 4 °C for further use. Flavonoid stocks were maintained at −20 °C.

### 4.7. Broth Micro-Dilution Assay

Inoculum preparation. Bacteria inoculums were obtained from suspended bacterial colonies in fresh Mueller-Hinton (MH) broth. The density of the bacteria culture required for the test was adjusted to 0.5 McFarland standard (1.0 × 10^8^ CFU/mL) in 10 mL MH broth using a turbidimeter (BioSan, Rīga, Latvia). The inoculum suspensions were divided in each well containing approximately 1 × 10^6^ CFU/well.

Extracts preparation. Ethanol and water extracts were dissolved in 95% ethanol and water, respectively. The stock extract solutions were prepared at a concentration of 100 mg/mL. Two-fold serial dilution was done in a 96-well microplate. The final concentrations of the extracts in each well were in the range of 9.78–250 mg/mL.

Amoxicillin, doxycycline, and gentamicin. Portions of 0.1 mg/mL were used as the standard controls for all experiments. The microplates were incubated at 37 °C for 24 h. The results were obtained by detecting the absorbance values at time intervals of 0, 18, and 24 h using a microplate reader (Tecan, Redwood City, CA, USA) at a wavelength of 625 nm. The bacterial growth curves were made and the inhibitory activities were then determined. The half maximal inhibitory concentration (IC_50_) values were calculated using CalcuSyn 2.1 software (Biosoft, Cambridge, UK), whereas the ninety-seven percent inhibitory concentration (IC_97_) values were calculated using CompuSyn 1.0 software (Combosyn Inc., Paramus, NJ, USA).

### 4.8. Analysis of Flavone Contents in *O. indicum* Extracts by HPLC

Quantitative analysis of the three flavones in the *O. indicum* extracts, namely, baicalin, baicalein and chrysin, was conducted using the HPLC method that was validated as described below.

High-performance liquid chromatographic analysis of baicalin, baicalein, and chrysin was performed using a Shimadzu LC-10ADVP system (Kyoto, Japan) equipped with a diode array detector (DAD) SPD-M10AVP and a column heater (Shimadzu, Kyoto, Japan). An X-terra C18 column (150 mm × 3.9 mm, 5 μm particle size) from Waters (Milford, MA, USA) was used. Gradient elution was performed with water–0.01% phosphoric acid (solvent A) and acetonitrile (solvent B) at a constant flow rate of 1.2 mL/min. Column temperature was 40 °C with an injection volume of 20 μL; detection was performed at 285 nm.

Linearity for standard baicalin, baicalein, and chrysin was determined by analysis of seven different concentrations, each injected in triplicate. Peak purity was investigated using a DAD for all peaks of interest. The precision of the method was evaluated by analyzing six independently prepared solutions of standard baicalin, baicalein, and chrysin on three different days. The peak area of each standard compound was determined and the relative standard deviation percent (%RSD) was calculated. The accuracy of the method was confirmed by determination of the recovery. The recovery of standard compounds was performed on samples spiked with three concentrations of each compound (10, 20, and 30 μg/mL). The validated HPLC analytical method was then applied for quantitative analysis of baicalin, baicalein, and chrysin in *O. indicum* fruit and seed extracts. System suitability of the analytical method, including tailing factor, theoretical plate, %RSD of peak area and SD of retention time, was also investigated.

## 5. Conclusions

From all of the results, *O. indicum* fruit and seed extracts showed significant in vitro antibacterial effects against four pathogenic bacteria, namely, *Staphylococcus intermedius*, *Streptococcus suis*, *Pseudomonas aeruginosa*, and *β-Escherichia coli*. These extracts, especially the extract from the fruits of *O. indicum* collected from Nakhon Pathom province (OFNE), could be major natural sources for development as a health supplement or herbal medicines in the future, particularly for the antibacterial effect to treat zoonotic diseases such as meningococcal meningitis and soft tissue bacterial meningitis. These flavonoids, namely, baicalin, baicalein, and chrysin, could be used as chemical markers for the quality control of *O. indicum* fruit and seed extracts. The relation between the flavonoid contents in the seed extracts and their antibacterial effects should be investigated. The optimized and validated HPLC method that was obtained in this study could be used for quantitative analysis of flavonoids in *O. indicum* fruit and seed extracts. Studies about the mechanism of action, toxicity, development of herbal formulation, and stability tests should be performed in the future.

## Figures and Tables

**Table 1:** Half maximal inhibitory concentrations (IC_50_) of *Oroxylum indicum* extracts and flavones.

Sample	IC_50_ (mg/mL)			
	*Staphylococcus intermedius*	*Streptococcus suis*	*Pseudomonas aeruginosa*	*β-Escherichia coli*
OFND	7.81 ^a^	>250 ^d^	>125 ^g^	>250 ^d^
OFNE	1.30	7.81 ^e^	39.20	66.85
OFCD	14.00	>250 ^d^	>250 ^d^	>250 ^d^
OFCE	1.96	7.81 ^f^	61.49	>250 ^d^
OSBD	1.95 ^b^	>250 ^d^	>250 ^d^	>250 ^d^
OSBE	>31.25 ^c^	>250 ^d^	527.01	>250 ^d^
Baicalin	0.36	0.53	0.22	0.33
Baicalein	2.05	>5 ^h^	0.17	0.43
Chrysin	>5 ^h^	>5 ^h^	0.34	1.56

Different letters in the same column are ^a^ At a concentration of 7.8 mg/mL, has a % Bacterial inhibition of 43.70%, ^b^ At a concentration of 1.95 mg/mL, has a % Bacterial inhibition of 23.77%, ^c^ The minimum inhibitory concentration is >31.25 mg/mL, ^d^ The minimum inhibitory concentration is >250 mg/mL, ^e^ At a concentration of 7.81 mg/mL, has a % Bacterial inhibition of 13.59%, ^f^ At a concentration of 7.81 mg/mL, has a % Bacterial inhibition of 2.18%, ^g^ The minimum inhibitory concentration is >125 mg/mL, ^h^ The minimum inhibitory concentration is >5 mg/mL. OFND, water extract from *O. indicum* fruits collected from Nakhon Pathom province; OFNE, ethanol extract from *O. indicum* fruits collected from Nakhon Pathom province; OFCD, water extract from *O. indicum* fruits collected from Chiang Rai province; OFCE, ethanol extract from *O. indicum* fruits collected from Chiang Rai province; OSBD, water extract from *O. indicum* seeds purchased from Bangkok; OSBE, ethanol extract from *O. indicum* seeds purchased from Bangkok.

**Table 2:** Ninety-seven percent inhibitory concentrations (IC_97_) of flavones in *O. indicum*.

Sample	IC_97_ (mg/mL)			
	*S. intermedius*	*S. suis*	*P. aeruginosa*	*β-E. coli*
Baicalin	2.48	12.43	1.83	2.31
Baicalein	20,413	-	5.99	1.86
Chrysin	-	-	1.81	169.86

- signifies no activity.

**Table 3:** Calibration data for baicalin, baicalein, and chrysin, including regression equations and correlation coefficient (*r*^2^).

Compound	Regression Equation	*r* ^2^
Baicalin	y = 42947.68x − 8583.02	0.9997
Baicalein	y = 64149.91x + 18864.34	0.9998
Chrysin	y = 53645.77x + 10586.07	0.9999

**Table 4:** Flavonoid contents in *O. indicum* extracts determined by high-performance liquid chromatographic (HPLC).

Sample	Content (% *w*/*w* in the Extract) *		
	Baicalin	Baicalein	Chrysin
OFND	0.19 ± 0.00 ^a^	0.14 ± 0.00 ^a^	0.02 ± 0.00 ^a^
OFNE	1.23 ± 0.01 ^b^	1.07 ± 0.02 ^b^	0.44 ± 0.00 ^b^
OFCD	0.37 ± 0.01 ^c^	0.14 ± 0.00 ^c^	0.02 ± 0.00 ^c^
OFCE	1.46 ± 0.01 ^d^	1.23 ± 0.01 ^d^	0.42 ± 0.01 ^d^
OSBD	9.03 ± 0.07 ^e^	1.27 ± 0.02 ^d^	0.96 ± 0.02 ^e^
OSBE	9.45 ± 0.13 ^f^	0.91 ± 0.02 ^e^	0.47 ± 0.01 ^f^

* Different letters in the same column are significantly different (*p* < 0.05). OFND, water extract from *O. indicum* fruits collected from Nakhon Pathom province; OFNE, ethanol extract from *O. indicum* fruits collected from Nakhon Pathom province; OFCD, water extract from *O. indicum* fruits collected from Chiang Rai province; OFCE, ethanol extract from *O. indicum* fruits collected from Chiang Rai province; OSBD, water extract from *O. indicum* seeds purchased from Bangkok; OSBE, ethanol extract from *O. indicum* seeds purchased from Bangkok.
